# Electrochemical Immunosensor for the Detection of Aflatoxin B_1_ in Palm Kernel Cake and Feed Samples

**DOI:** 10.3390/s17122776

**Published:** 2017-11-30

**Authors:** Farah Asilah Azri, Jinap Selamat, Rashidah Sukor

**Affiliations:** 1 Food Safety and Food Integrity (FOSFI), Institute of Tropical Agriculture and Food Security, Universiti Putra Malaysia, 43400 Serdang, Selangor, Malaysia; farah.asilah90@gmail.com (F.A.A.); rashidah@upm.edu.my (R.S.); 2 Department of Food Science, Faculty of Food Science and Technology, Universiti Putra Malaysia, 43400 Serdang, Selangor, Malaysia

**Keywords:** PKC, feed, electrochemical immunosensor, AFB_1_, ELISA, nanocomposite

## Abstract

Palm kernel cake (PKC) is the solid residue following oil extraction of palm kernels and useful to fatten animals either as a single feed with only minerals and vitamins supplementation, or mixed with other feedstuffs such as corn kernels or soy beans. The occurrence of mycotoxins (aflatoxins, ochratoxins, zearalenone, and fumonisins) in feed samples affects the animal’s health and also serves as a secondary contamination to humans via consumption of eggs, milk and meats. Of these, aflatoxin B_1_ (AFB_1_) is the most toxically potent and a confirmed carcinogen to both humans and animals. Methods such as High Performance Liquid Chromatography (HPLC) and Liquid Chromatography–Mass Spectrometry (LC-MS/MS) are common in the determination of mycotoxins. However, these methods usually require sample pre-treatment, extensive cleanup and skilled operator. Therefore, in the present work, a rapid method of electrochemical immunosensor for the detection of AFB_1_ was developed based on an indirect competitive enzyme-linked immunosorbent assay (ELISA). Multi-walled carbon nanotubes (MWCNT) and chitosan (CS) were used as the electrode modifier for signal enhancement. *N*-ethyl-*N*′-(3-dimethylaminopropyl)-carbodiimide (EDC) and *N*-hydroxysuccinimide (NHS) activated the carboxyl groups at the surface of nanocomposite for the attachment of AFB_1_-BSA antigen by covalent bonding. An indirect competitive reaction occurred between AFB_1_-BSA and free AFB_1_ for the binding site of a fixed amount of anti-AFB_1_ antibody. A catalytic signal based on horseradish peroxidase (HRP) in the presence of hydrogen peroxide (H_2_O_2_) and 3,3′,5,5′-tetramethylbenzidine (TMB) mediator was observed as a result of attachment of the secondary antibody to the immunoassay system. As a result, the reduction peak of TMB_(Ox)_ was measured by using differential pulse voltammetry (DPV) analysis. Based on the results, the electrochemical surface area was increased from 0.396 cm^2^ to 1.298 cm^2^ due to the electrode modification with MWCNT/CS. At the optimal conditions, the working range of the electrochemical immunosensor was from 0.0001 to 10 ng/mL with limit of detection of 0.1 pg/mL. Good recoveries were obtained for the detection of spiked feed samples (PKC, corn kernels, soy beans). The developed method could be used for the screening of AFB_1_ in real samples.

## 1. Introduction

**Figure 1:**
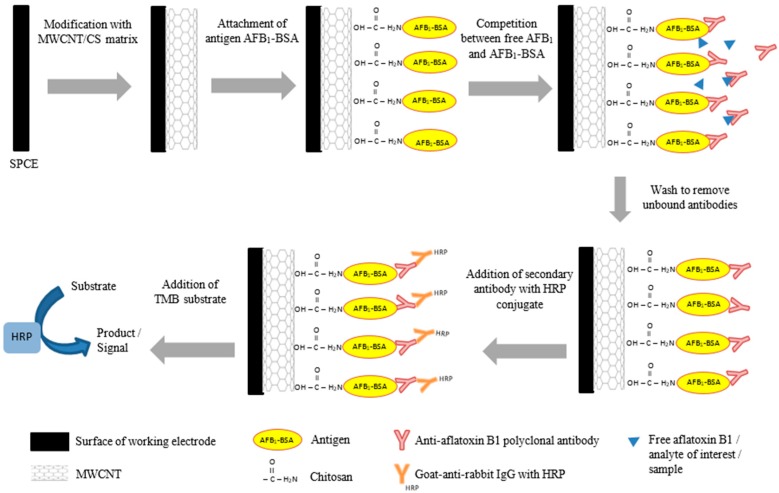
Fabrication of the electrochemical immunosensor by immobilization of the optimized indirect competitive enzyme-linked immunosorbent assay (ELISA) onto the modified electrode (multiwalled-carbon nanotubes/chitosan/screen-printed carbon electrode, MWCNTs/CS/SPCE).

To lessen the feeding cost of livestock, which competes directly with humans for the same food crops, attempts have been made to use alternative sources of protein and energy. These have mostly been of agro-industrial byproduct origin, which is not directly utilizable by human. Malaysia is one of the world’s leading producers of palm oil, and the availability of palm is therefore, extensive. Palm kernel cake (PKC), as shown in Figure 1, is a byproduct of oil extraction from palm [1]. PKC is also known as palm kernel meal (PKM) or palm kernel expeller (PKE). PKC is either produced from expeller extraction or through solvent extraction [2]. In terms of quality, there is no significant difference between expeller-extracted and solvent-extracted PKC, although expeller-extracted PKC might contain higher oil content (4–8%) as compared to solvent-extracted PKC (1–2%). As a feed, PKC provides protein, energy, minerals and vitamins. According to Idrus (2016), over two million tons of PKC is produced annually by Malaysia, of which 94% is exported, leaving about 120,000 tons for local consumption [3]. This only proves that there is a necessity for maximum utilization of the limited PKC supply. Inappropriate handling during production and storage are the contribution factors towards mycotoxin contamination in feeds. This has drawn public attention, especially the concern on human and animal health, not to mention the huge waste of the limited PKC supply [4]. Mycotoxin contamination of feeds can occur in the field, during harvesting, drying, transportation and even storage. Mycotoxin production is largely influenced by the environmental conditions including temperature, humidity [5] and agronomic practices [6]. This has culminated into the need for the detection of mycotoxins in PKC. Mycotoxins are generally chemically stable; hence, they can endure storage and processing [7]. The best strategy to control consumption of mycotoxins is only by prevention; discarding the moldy feed. However, since mycotoxins are very stable and can survive long after the initial contaminant has disappeared, the absence of mold does not ensure the crop or crop product is mycotoxin-free [8]. In addition, several mycotoxins have a high carryover rate and will present in the livestock produce such as milk [9]. This can subsequently result in secondary contamination of humans.

PKC has been widely used in the international feed market as a major component for poultry feed ingredients. Yibadatihan et al. (2014) did a simultaneous detection of multi-mycotoxins in palm kernel cake (PKC) using Liquid Chromatography–Mass Spectrometry/ Mass Spectrometry (LC-MS/MS) [4]. Based on the results, 92% of the 25 samples were detected to contain AFB1 (1.76–18.99 µg/kg). Since Malaysia experiences tropical climate with high temperature ranging from 24 to 32 °C, rainfall throughout the year and relative humidity in the range of 70–80% during wet season and 50–60% during dry season [10,11], crop commodities such as PKC are naturally exposed to fungal infection and the subsequent mycotoxin production.

Among the major mycotoxins contaminating foods and feeds are aflatoxins which are primarily hepatotoxic to animals as well as reducing the production of milk, eggs and weight gain [12]. Aflatoxins are predominantly synthesized by *Aspergillus flavus* and *A. parasiticus* [13]. Of the four major aflatoxin metabolites (AFB_1_, AFB_2_, AFG_1_, and AFG_2_), only AFB_1_ has regulatory limits in animal feed as prescribed by the European Union [14].

New methods for detecting mycotoxins are presently being studied and developed to enhance the accuracy and range of mycotoxins to be tested, including qualitative and quantitative techniques. Enzyme-linked immunosorbent assay (ELISA) is one of these techniques, and uses antibodies to detect target molecules (antigens). ELISA is also highly sensitive, easy to perform, and frequently applied in biosensor [15]. Biosensors can be useful as an alternative method for mycotoxin determination due to their unique properties of real-time, less laborious, shorter time and minimal cost [16]. Although promising, the adaptation of ELISA into an electrochemical approach is not always forthright since several factors should considered to achieve performances comparable or even better than that of conventional methods [17].

In the present work, we have successfully developed a sensitive immunosensor for the detection of AFB_1_ in PKC and other feed samples (corn kernels and soy beans). We designed an electrochemical immunosensor based on an indirect competitive ELISA. The optimized ELISA was transferred onto the modified screen-printed carbon electrode (SPCE) with multi-walled carbon nanotubes (MWCNTs) and chitosan (CS). The use of nanomaterials in sensor development is believed to provide good biocompatibility for biological components and subsequently enhance the electrochemical performance as well as increase the sensitivity of the respective sensor. To the best of our knowledge, this is the first report on the detection of AFB_1_ in PKC and feed samples by using electrochemical immunosensor. This technique can be a prevailing immunosensor for AFB detection in the upcoming and might further provide a handy tool to guarantee food safety.

## 2. Materials and Methods

### 2.1. Chemicals and Reagents

AFB_1_-BSA, rabbit anti-AFB_1_, goat anti-rabbit IgG horseradish peroxidase (HRP) conjugate, 3,3′,5,5′-tetramethylbenzidine (TMB) substrate, carbonate-bicarbonate buffer (capsule), multi-walled carbon nanotubes (MWCNTs), medium molecular weight chitosan (CS) and potassium ferricyanide, K_3_[Fe(CN)_6_], were purchased from Sigma-Aldrich (St. Louis, MO, USA). Aflatoxin B_1_ (AFB_1_) standard solution (20 µg/mL in methanol) was purchased from Supelco Analytical (Bellefonte, PA, USA). Skimmed-milk powder was obtained from Nacalai Tesque (Kyoto, Japan). Other reagents were of analytical grade, and all aqueous solutions were prepared using deionized water. Phosphate buffer saline (PBS) was prepared at 10× stock concentration and diluted to 1×. Tween 20 (0.05%) was added to 1 L of 1× PBS to make PBST as a washing buffer in ELISA. For supporting electrolyte, 0.17 g K_3_[Fe(CN)_6_] was added into 100 mL of 1× PBS to make a 5 mM solution.

### 2.2. Apparatus

All the electrochemical measurements including cyclic voltammetry, differential pulse voltammetry and chronoamperometry were carried out using a portable µSTAT 400 Bipotentiostat/Galvanostat, and the current was analyzed by Dropview Software by Dropsens. All measurements were done using disposable screen-printed carbon electrodes (SPCE) from Dropsens. The SPCE is made up of ceramic (L33 × W10 × H0.5 mm) with silver electric contacts. It consists of three types of electrodes: working (carbon, 4 mm Ø), counter (carbon) and reference (silver).

### 2.3. Modification of the Working Electrode

The modification of working electrode was performed based on procedures described by Azri et al. (2017). The SPCE was modified by using nanocomposite of MWCNTs/CS. The pristine MWCNTs were first functionalized by using H_2_SO_4_:HNO_3_ (3:1) treatment to modify the surface of carbon nanotubes with carboxylic acid groups. Five milligrams of MWCNTs powder was dispersed into 1 mL of 0.5% chitosan solution (prepared in 1% acetic acid). Then, the mixture was sonicated for 1 h to produce uniform black suspension. Then, 10 µL of the dispersion was added to working electrode and dried at 60 °C in an oven for 10 min to obtain MWCNTs/CS/SPCE.

### 2.4. Design of Electrochemical Immunosensor

Prior to transferring the optimized indirect competitive ELISA onto the MWCNTs/CS/SPCE, 20 µL of activated fluid containing 0.4 M of *N*-ethyl-*N*′-(3-dimethylaminopropyl)-carbodiimide (EDC) and 0.2 M of *N*-hydroxysuccinimide (NHS) at EDC:NHS (2:1 *v*/*v*) was added to the electrode surface for 1 h at room temperature to activate carboxylic acid groups. Then, MWCNTs/CS/SPCE was washed thoroughly using PBS. The competitive ELISA was done by coating the electrode with 0.25 µg/mL of AFB_1_-BSA. The SPE was left to dry for 30 min at room temperature before blocked with 8% skimmed-milk in PBS. AFB_1_ standards (10 µL), ranging from 10^−4^ to 10^3^ ng/mL in 10% methanol, and 10 µL of rabbit anti-AFB_1_ (1:5000, *v*/*v*) was added simultaneously onto the MWCNTs/CS/SPCE. AFB_1_ standard was allowed to compete with the coating conjugate (AFB_1_-BSA) for antibody binding site for 30 min at room temperature. Then, goat anti-rabbit HRP conjugate was added at (1:5000, *v*/*v*) and the electrode was incubated for another 30 min at room temperature. Figure 1 shows the schematic diagram of the electrochemical immunosensor design.

### 2.5. Electrochemical Reaction

Aqueous hydrogen peroxide (H_2_O_2_) was reduced by the HRP which conjugated on the secondary antibody. Then, HRP_(Ox)_ was revived to HRP_(Red)_ with aid of the mediator, TMB_(Red)_ via chemical oxidation to TMB_(Ox)_. TMB was used as the electroactive mediator since it can be reduced directly on the surface of electrode. Subsequently, the reduced TMB increased the reduction current.

### 2.6. Sample Preparation

Fourteen samples were used to validate the developed immunosensor. The samples consisted of eight PKC samples which were collected from different factories and six various formulations of PKC mixed with other feeds including corn kernels and soy beans. Five grams of the PKC and feed samples was spiked with 500 µL AFB_1_ standard in methanol (10 ng/mL). The samples were mixed using vortex for 1 min. Next, 25 mL of extraction solvent (85% methanol:15% PBS) was added and agitated in shaker for 1 h at 100 rpm at room temperature. The mixture was then centrifuged at 6000 rpm for 10 min. The supernatant (1 mL) was collected. The supernatant was diluted three times (1:2, *v*/*v*) with PBS.

### 2.7. Method Validation

An indirect competitive ELISA was performed based on the optimized conditions. Microtiter plates were coated with AFB_1_-BSA at 0.25 µg/mL and were incubated at 4 °C overnight. The plates were blocked with 1% BSA in PBS for 1 h at room temperature with gentle shaking. Peanut extract (50 µL) and primary antibody (50 µL; 1:5000, *v*/*v*) were added simultaneously into the wells. The sample was allowed to compete with the coating conjugate (AFB_1_-BSA) for antibody binding site for 1 h at room temperature. Then, 100 µL of the secondary antibody (1:5000, *v*/*v*) was added. The plates were incubated for another 1 h at room temperature. TMB substrate was added and incubated for another 30 min in the dark at room temperature until blue color developed. The reaction was stopped by adding 1N H_2_SO_4_, and absorbance was read at 450 nm. The plate was washed using PBS-T between each step for three times.

## 3. Results and Discussion

### 3.1. Optimization of Conditions for Electrochemical Detection

**Figure 2:**
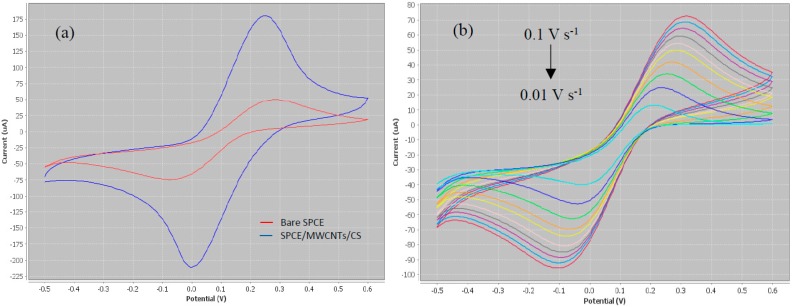
CV characteristics of: (**a**) screen-printed carbon electrode modified with MWCNTs and CS in comparison with bare electrode; and (**b**) modified electrode under different scan rate in a range of 0.01 to 0.1 V s^−1^. The analysis was done in 0.1 M PBS with 5 mM of K_3_[Fe(CN)_6_]. Scan was set to three cycles from −0.5 to 0.6 V (vs. Ag/AgCl).

In the present work, optimization of the physical parameters including coating volume, coating ratio, drying condition and pH of buffer for electrode modification is not described in detail, as it was performed as described in our previous work [18]. However, the electrochemical conditions had to be re-optimized since different type of potentiostat (portable) was used. The electrochemical properties of the modified electrode were characterized by cyclic voltammetry (CV) analysis, as shown in Figure 2a.

From the CV results, a pair of well-defined reversible redox peaks corresponding to [Fe(CN)_6_]^3−^ which was present as the electroactive species was clearly visible. SPCE modified with the layer of MWCNTs/CS showed a clear enhancement compared to bare electrode. This was due to the excellent properties of carbon nanotubes, which can intensely promote the sensing capacity as well as enhance the electron transfer and subsequently increase the sensitivity. The electroactivity of CNTs was due to the presence of reactive groups on the surfaces [19]. However, in the pristine state, nanotubes were not well dispersed in the organic matrices and, therefore, suitable improvement of the surfaces was required [20]. In the present work, the pristine MWCNTs were functionalized using harsh acid treatment to introduce carboxylic acid (–COOH) groups onto their surfaces. The presence of –COOH groups provided a convenient site for protein immobilization.

The intensity of the redox current obtained on the MWCNTs/CS/SPCE increased by three times compared to the bare electrode (calculated from corresponding curves in Figure 2a. Sulfonation of the MWCNTs improved the electrical conductivity as well as the dispersion of material in the matrix [21]. The treated MWCNTs prevented agglomeration, and formed stronger hydrogen bonding between the carbon nanotubes. The real active surface area of the electrode was calculated using the Randles–Sevcik Equation:

*i*_p_ = (2.69 × 10^5^) *n*^3/2^ ACD^1/2^*v*^1/2^ … (1)

where *n* is the number of electrons, A is the electrode area (cm^2^), C is the concentration (mol cm^−3^), D is the diffusion coefficient (cm^2^ s^−1^), and *v* is the scan rate (V s^−1^). Based on Equation (1), it was calculated by substituting *n* = 1 (in the Fe(CN)_6_^3−^ system), A = πr^2^ = (22/7)(0.2 cm)^2^ = 0.126 cm^2^, C = 5.0 × 10^−3^ mol L^−1^, D = 7.6 × 10^−6^ cm^2^ s^−1^ and *v* = 0.1 V s^−1^. The geometrical surface area of the electrode was 0.126 cm^2^. As expected, the real electrochemical surface area was much larger. Active surface area of bare SPCE was calculated to be 0.396 cm^2^ and it increased to 1.298 cm^2^ after modification with MWCNT treated with H_2_SO_4_ + HNO_3_.

Figure 2b shows the effect of scan rate in a range of 0.01–0.1 V s^−1^ on CVs of the MWCNTs/CS/SPCE. Based on the voltammograms, the peaks increased with the increasing scan rate. The intensities of the redox currents proved a fast electrochemical process by the diffusion of ferricyanide, and no definitive adsorption of ferricyanide on the surface of corresponding electrode [22]. The diffusion coefficient was calculated using Equation (1) for each scan rate to measure the quantity of a substance in diffusing from one region to another. From the result, 0.05 V s^−1^ was chosen as the optimum scan rate due to small ip_a_/ip_c_ ratio and high diffusion coefficient. Based on the electrochemical reaction described in Section 2.5, the redox profile of TMB/H_2_O_2_ was investigated without the presence of electroactive species. One reduction peak was observed within the potential range of 0.2 to 0.4 V, as a result of HRP activity which was generated by TMB_(Ox)_. Therefore, the work was furthered in differential pulse voltammetry (DPV) analysis by narrowing the suitable detection potential for the HRP activity.

### 3.2. Repeatability and Reproducibility of the Modified Electrode

The repeatability and reproducibility are important factors in developing a biosensor to ensure stability and reliability of the support medium. In the present work, the repeatability of the electrode showed that the current reduced when the number of scans increased. The scan of the same modified electrode was done up to five times and resulted in a relative standard deviation (RSD) of 7.58%. The reproducibility of the modified electrode was determined by five manually parallel-made MWCNT/CS/SPCEs. The result showed an acceptable 6.4% (*n* = 5) showing a reliable modification strategy of an electrode.

### 3.3. Detection of Aflatoxin B_1_ by Differential Pulse Voltammetry Analysis

An indirect competitive ELISA was done using eight serial dilutions of AFB_1_ standard solution with concentration in the range of 0.0001 to 1000 ng/mL. The assay was done similar to microtiter plate. However, the volume of the reagent used to coat the surface of electrode was adjusted from 100 µL to 10 µL due to smaller diameter of working electrode. This is one of the advantages in developing immunosensor which requires lesser reagent and subsequently lowers the production cost. Furthermore, since the volume used was reduced, the reaction time was also reduced. Thirty minutes of incubation was sufficient to allow the binding to take place as compared to 1 h in microtiter plate. The modified electrode (MWCNTs/CS/SPCE) was activated by EDC/NHS coupling agent and formed a stable matrix for antibody attachment. The competition of the targeted analyte with the primary antibody (anti-aflatoxin B_1_) occurred when added to the system simultaneously (without pre-incubation).

**Figure 3:**
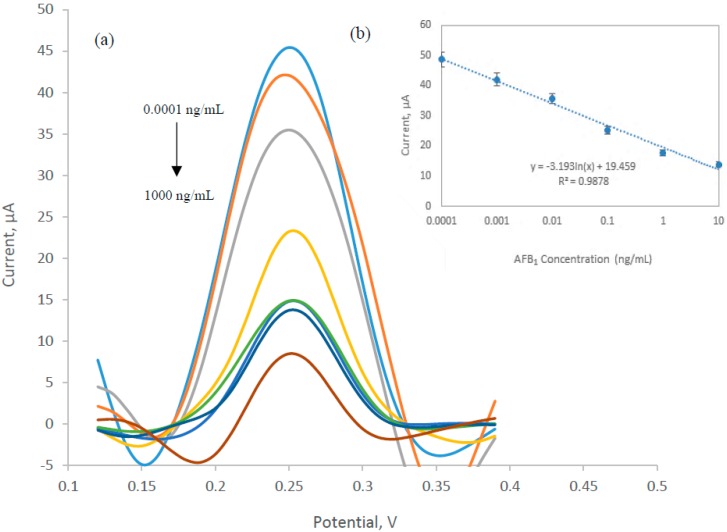
(**a**) Differential pulse voltammetry (DPV) analysis within the potential range of 0.1 to 0.4 V using TMB and 0.06% H_2_O_2_ as substrate. Detection of AFB_1_ was based on the reduction of TMB_(Ox)_ by HRP enzyme. The current peaks were found at 0.25 ± 0.01 V. (**b**) Linear regression of standard curve for AFB1 within the range of 0.0001 to 10 ng/mL.

Figure 3a shows the current response based on the enzyme activity of HRP, in which increasing AFB_1_ concentrations resulted in a decrease in current peak. This is because when higher concentration of AFB_1_ was present in the system, it fully occupied the binding site of the available antibody and therefore being eliminated from the system during the washing step. Linear regression plot in Figure 3b represents the linear working range of AFB_1_ determination in the range of 0.0001 to 10 ng/mL (*R*^2^ = 0.9878). The limit of detection (LOD) referred to the concentration corresponding to the f(*x*) value obtained by subtracting three times the standard deviation of the zero point from the mean of the zero standard measurement (mean value 3 s). The LOD of the developed system was calculated to be at 0.1 pg/mL. Zhang et al. developed an electrochemical immunosensor based on single-walled carbon nanotubes/chitosan which could quantitatively detect AFB_1_ from 0.01 to 100 ng/mL with a detection limit of 3.5 pg/mL [23]. The MWCNTs used in this study are electrochemically more stable and could provide larger surface area for electron transfer to take place and subsequently enhance the sensitivity and catalytic activity [24,25].

### 3.4. Calibration Curve of Spectrophotometric ELISA

**Figure 4:**
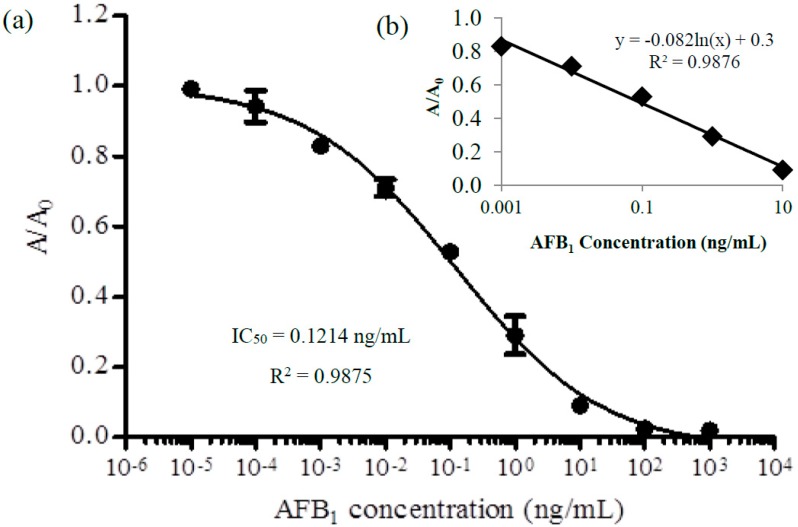
(**a**) Calibration curve of AFB_1_ for indirect ELISA format using spectrophotometric detection (Error bar = standard deviation, *n* = 3); and (**b**) linear regression of standard curve with AFB_1_ working range from 0.001 to 10 ng/mL.

The data were collected using the optimal assay conditions described in Section 2.7. AFB_1_ standard was used to plot a standard curve for the quantification of analyte in the sample. The competitive standard curve was inversely proportional between the correlation signals found and the analyte concentrations. The response of the signal was relative to the amount of enzyme conjugate bound to the support. This was due to the competition of analyte and enzyme conjugate to the antibody. In Figure 4a, a nonlinear calibration curve was obtained. The A/A_0_ reading decreased with the increase of concentration of AFB_1_ standard solution. Based on the result, higher signals were observed from 0.0001 to 0.001 ng/mL, then dramatically decreased and declined after 100 ng/mL. High signals indicated that high amount of primary antibody was bound to the antigen coated in the wells, which meant that no or less free analyte (AFB_1_ standard) was present. From the observation, the working range was between 0.001 to 10 ng/mL of AFB_1_ (*R*^2^ = 0.9876). Figure 4b shows the linear plot within the working range to obtain the equation for this assay. The calibration curve was fitted into a nonlinear regression using the four-parameter logistic as in Equation (2):

(2) $$F\left(x\right)=d+\frac{a-d}{1+{\left(\frac{x}{c}\right)}^{b}}\;\ldots$$

where *a* and *d* are the minimum and maximum asymptote of calibration curve, respectively; *c* is the inflection point; and *b* is the hill slope. EC_50_ differs from IC_50_, in which EC_50_ refers to the effective concentrations that gives half-maximal response, while IC_50_ is the concentration of an inhibitor where the response or binding is reduced by half [26]. Therefore, LOD was calculated based on mean ±3 SD. The value was inserted into the GraphPad Prism software and the interpolated *x*-value was obtained. Based on the value, the LOD was found to be 0.15 ng/mL, which was in the range of maximum level of AFB_1_ (20 ng/mL).

### 3.5. Test on PKC and Feed Samples

The feasibility test of the developed immunosensor was then conducted in PKC and feed samples (corn kernels, soy beans, mixtures). Sample extracts were used for the analysis to replace PBS containing AFB_1_ standard. The samples were spiked with respective AFB_1_ standard solution and the extraction was done as previously described. The extract of food samples especially in solid-phase contains several matrix components which can affect the results due to the matrix effect. Matrix interference is one of the challenges in immunoassay for food analysis which can cause false positive [27]. The enzyme activity and the interaction between the antigen/analyte with the antibody will be hindered due to matrix interference. However, this problem can be minimized by diluting the sample extract or cleanup procedures using solid-phase extraction. The PKC and feed sample extracts were diluted using PBS and the original concentration in the diluted sample was calculated according to Equation (3):

(3) $${\mathrm{AFB}}_{1}\left(\mathrm{ng}/g\right)=\left\{\frac{\left[{\mathrm{AFB}}_{1}\left(\mathrm{ng}/\mathrm{mL}\right)\;\mathrm{in}\;\mathrm{sample}\;\mathrm{extract}\right]\left[\mathrm{solvent}\;\mathrm{extract}\;\mathrm{volume}\right]}{\mathrm{sample}\;\mathrm{weight}}\right\}\times \mathrm{dilution}\;\mathrm{factor}$$

where the solvent extract volume is 10 mL, sample weight is 5 g and the dilution factor is 3. By substituting the values in Equation (2), the concentration of AFB_1_ (ng/g) in real sample can be directly calculated by multiplying the concentration of AFB_1_ (ng/mL) by 6. The mean ± SD of each sample and recovery of each spiked sample were calculated and the values are reported in Table 1. The results show that there is a good agreement between the results obtained from electrochemical ELISA (immunosensor) when compared to spectrophotometric ELISA. Moreover, the recovery of AFB_1_ for spiked sample analysis is in the range of 78–117%. These indicated that the method has a good accuracy and precision. Based on the results, it was found that samples with combination of three different feeds (corn kernels + soy beans + PKC) showed greater matrix effect compared to the extraction of a single feed material. Therefore, the samples were diluted further to reduce the matrix effect.

## 4. Conclusions

An electrochemical immunosensor for ultrasensitive detection of AFB_1_ based on MWCNT/CS composite film has been developed in the present work. The experiments have demonstrated that MWCNT could remarkably enhance the electron transfer rate between the immunosensor and the redox in the solution due to their unique electrochemical properties, and subsequently increased the sensitivity. Immobilization of antigen (AFB_1_-BSA) onto the nanocomposite film through covalent bonding improved the electrochemical stability of the developed sensor and ensured a stable binding of antibody onto the assay. The developed immunosensor provided good sensitivity, precision, reproducibility and acceptable accuracy for sample determination, which proved it as a promising sensor. It also showed a dynamic linear range from 0.0001 to 10 ng/mL with the limit of detection calculated to be 0.1 pg/mL.

## Figures and Tables

**Table 1:** Comparison of AFB_1_ in spiked feed samples between electrochemical ELISA and spectrophotometric ELISA.

Feed Samples Extract/Detection of AFB_1_	Electrochemical ELISA			Spectrophotometric ELISA
	AFB_1_ conc. (ng/mL)	Blank (ng/mL)	Recovery (%)	Spiked-Blank (ng/mL)
Corn kernels	9.679	0.043	96.4	8.981
Soy beans	8.428	0.310	81.2	12.035
Corn kernels + soy beans	11.001	0.643	103.6	12.760
Corn kernels + soy beans + 70% PKC	9.520	0.383	91.4	8.554
Corn kernels + soy beans + 30% PKC	7.864	0.043	78.2	7.498
Corn kernels + soy beans + 20% PKC	7.981	0.176	78.0	9.003
PKC sample 1	11.019	0.245	107.7	8.765
PKC sample 2	8.855	0.165	86.9	8.808
PKC sample 3	8.171	0.411	77.6	8.087
PKC sample 4	10.669	0.333	103.4	10.617
PKC sample 5	9.575	0.108	94.7	9.035
PKC sample 6	10.788	0.299	104.9	11.918
PKC sample 7	11.141	0.423	107.2	9.663
PKC sample 8	11.905	0.165	117.4	10.321
